# Implications of Indirect Biomarkers of Intestinal Permeability in the Stools of Newborns and Infants with Perinatal Risk Factors for Intestinal Colonization Disorders and Infant Feeding Patterns

**DOI:** 10.3390/nu14112224

**Published:** 2022-05-26

**Authors:** Diana Sochaczewska, Maciej Ziętek, Barbara Dołęgowska, Agnieszka Kordek, Małgorzata Szczuko

**Affiliations:** 1 Department of Neonatology, Pomeranian Medical University in Szczecin, 72-009 Police, Poland; diana.sochaczewska@pum.edu.pl (D.S.); agnieszka.kordek@pum.edu.pl (A.K.); 2 Department of Perinatology, Obstetrics and Gynecology Pomeranian Medical University in Szczecin, 72-009 Police, Poland; 3 Immunology and Laboratory Medicine, Department of Microbiology, Pomeranian Medical University in Szczecin, 71-899 Szczecin, Poland; barbara.dolegowska@pum.edu.pl; 4 Department of Human Nutrition and Metabolomics, Pomeranian Medical University in Szczecin, 71-460 Szczecin, Poland

**Keywords:** LPS, occludin, zonulin, intestinal permeability, route of delivery, antibiotic therapy, breastfeeding

## Abstract

Background: The intestinal microbiota of pregnant women and factors disturbing the microbial balance of their gastrointestinal tract during the perinatal period may be the cause of dysbiosis and thus intestinal permeability syndrome in their children. The purpose of this study was to analyze the implications of intestinal permeability parameters in the stools of newborns and infants with perinatal risk factors for intestinal colonization disorders (the route of delivery, antibiotic therapy in the neonatal period and the abandonment of breastfeeding). Methods: The study included 100 mother–child pairs. All children were born from uncomplicated and term pregnancies (between 37 and 42 weeks of gestation). In order to determine the parameters of dysbiosis and intestinal permeability, we determined the concentrations of zonulin and occludin in stool samples taken from all children at 0 (i.e., at birth), 3, 6 and 12 months of age. Elevated levels of lipopolysaccharide (LPS) are associated with metabolic diseases and its presence may be indicative of TJ injury and the onset of leaky gut syndrome. To indirectly determine the presence of endotoxemia, the concentrations of lipopolysaccharide were also measured in stool samples taken from all children at 0, 3, 6 and 12 months of age. We analyzed the relationship between the markers studied and perinatal risk factors for impaired intestinal colonization, including the mode of delivery, the method of feeding, and a family history of allergy. Results: During the first 3 months of infant life, higher concentrations of fecal occludin and zonulin were most often accompanied by higher values of fecal LPS. Similarly, higher concentrations of zonulin were accompanied by higher values of occludin. There were no significant differences in the stool concentrations of the studied markers during the first year of life between children born by caesarean section and those born naturally. In addition, the method of feeding had no significant effect on the changes in the concentrations of the determined fractions. Antibiotic therapy was associated only with an increase in the fecal occludin concentration after birth, without any effect on zonulin, occludin or LPS levels. The use of probiotic therapy in infants resulted in a decrease in only LPS concentrations at 3 months of age, with no effect on zonulin or occludin concentrations at 0, 6 and 12 months. Conclusions: Perinatal factors related to intestinal permeability are important during the first 3 months of infant life. However, we found that the mode of delivery had no influence on the parameters of infant intestinal leakage during the first year of life. In addition, the mode of infant feeding—breast or exclusively formula—did not significantly affect the changes in the concentrations of LPS, zonulin or occludin in the stools of children. A short-term increase in occludin concentrations after delivery in the stools of children from mothers undergoing antibiotic therapy indicates a negative but reversible influence of intrapartum antibiotics on the intestinal integrity of children in the perinatal period. Probiotic therapy seems to have a positive effect on reducing endotoxemia in children during the first 3 months of life. The presence of LPS at 3 months did not affect intestinal tightness at any of the later measured periods of the infants’ lives.

## 1. Introduction

The development of the fetal gastrointestinal microbiota begins during intrauterine life, and a disturbed microbial balance in the maternal gastrointestinal tract may be the cause of dysbiosis in the yet unborn child. According to current reports, some of the most important causes of impaired colonization are the route of delivery, the administration of antibiotic therapy during the neonatal period and a lack of breastfeeding [1]. Changes in the composition of the gut microbiome in early life may be associated with several chronic disorders in infants [2]. According to Langherdries et al. [3], the postnatal contact of the neonate with an invasive microbial environment triggers immunological mechanisms within the intestinal tissues. The modification of the quality of the host bacteria and their interactions leads to the impaired colonization of the intestine, which favors the acquisition of abnormal immune defenses in the neonate and, consequently, the appearance of autoimmune and allergic diseases later in life [2]. Physiologic delivery is a continuation of the already initiated intrauterine colonization of the fetus, occurring through the contact of the newborn with the rectovaginal flora of the mother [4]. In this period, antibiotic therapy is a factor that significantly disturbs the quality and quantity of colonizing bacteria [1]. By the end of the first month of life, differences in the type of bacterial colonies are noticeable, depending on the neonate’s diet. The quality of the intestinal microbiota determines the appropriate type of immunological response and the tightness of intercellular connections in the intestinal epithelium. One of the basic types of intercellular junctions within the intestine is tight junctions (TJs). TJs are multiprotein complexes of integral membrane proteins (claudins and occludins) and cytoplasmic scaffolding proteins (ZO-1, ZO-2 and ZO-3) [5]. They are the most important regulatory element of intestinal permeability, and they maintain cell polarity by limiting the movement of proteins across the cell membrane. Disturbed intestinal colonization can damage the intestinal barrier by disrupting the expression and function of TJ building proteins, resulting in the unsealing of intercellular junctions. There is evidence suggesting that the disruption of the intestinal epithelial barrier increases the movement of bacteria and bacteria-related products across the epithelium [6]. As a consequence, dysbiosis in the early neonatal period may result in so-called leaky gut syndrome and promote the development of food allergies, recurrent infections and autoimmune diseases, including irritable bowel syndrome, Hashimoto’s disease, obesity, asthma and diabetes [7]. According to recent reports, a reduction in the expression of TJs in some neurodegenerative diseases (e.g., Parkinson’s disease) is linked with increased intestinal permeability, i.e., leaky gut syndrome [8].

Indirect markers of impaired intestinal function and excessive permeability include zonulin (ZO) and endotoxin. ZO is an endogenous protease that is secreted by intestinal epithelial cells and hepatocytes. It has been shown to activate the ZOT (zonula occludens toxin) receptor. ZOT is an enterotoxin released by *Vibrio cholerae* that recognizes non-native ZOT conformers and localizes to intercellular contacts [9]. Referred to as the gate of the gut, ZO causes the reversible disassembly of the intercellular tight junctions within the intestine. Occludin and ZO proteins act together to determine the structural organization of TJs and form a classical barrier to the diffusion of solutes through the paracellular pathway. They regulate the transport of ions, macromolecules and immune cells. The increased secretion of ZO causes the reversible unsealing of tight junctions between cells and, consequently, an increase in intestinal barrier permeability [5].

Elevated levels of ZO in blood, urine or fecal samples are observed in individuals suffering from certain autoimmune diseases, especially those with an inflammatory component, including diabetes, celiac disease, obesity and RA. Gliadins—present in wheat, as well as rye and barley proteins are one of several dietary factors that can activate intestinal ZO receptors, resulting in increased intestinal permeability. Equally important for intestinal mucosal permeability is the balance of natural bacterial flora [10].

A disruption of the intestinal bacterial flora in favor of Gram-negative bacteria leads to the production of undesirable bacterial metabolites, which are also responsible for leaky gut symptoms. Lipopolysaccharide endotoxin (LPS), also called bacterial endotoxin, can be used to assess these changes. The presence of LPS during acute and chronic inflammatory reactions leads to the activation of immune cells and cytokines [6]. Under normal healthy conditions, LPS is eliminated from the body via HDL, whereas in diseased patients, LPS is redistributed by LDL and VLDL lipoproteins. An increase in circulating LPS in the organism results in an increase in glucose, triglycerides and inflammatory markers, as well as an increase in insulin resistance [11].

Various serum biomarkers are used in the assessment of intestinal permeability, including zonulin, calprotectin, fatty acid-binding proteins, citrulline, glucagon-like peptide (GLP)-2, LPS, LPS-binding protein (LBP) and serum amyloid A, in addition to fecal markers of intestinal permeability and markers of intestinal inflammation, e.g., alpha (α)-1-antitrypsin (AAT) and lipocalin 2 (LCN2), among others [12]. However, there are few studies in the literature on the use of occludin, zonulin and LPS in the assessment of intestinal permeability in children, determined based on the feces of children in association with perinatal factors.

## 2. Materials and Methods

### 2.1. Study Group

The study design was that of a prospective cohort study, characterized by cohort selection and the assessment of risk factors associated with indirect biomarkers of intestinal permeability in the stools of newborns and infants. The study was conducted in three primary steps: identification of participants, observation of the group over time to assess the dynamics of changes in the concentrations of the stool biochemical parameters studied, and the comparison of results at intervals. From a population of 1570 women giving birth in the Department of Perinatology, Obstetrics and Gynecology in Szczecin, 100 mother–child pairs were included in the study. Every second mother–child pair meeting the inclusion criteria and whose delivery took place between 1 January 2021 and 31 December 2021 was recruited. Randomization of participants in the trial was conducted by a team of investigators. The sampling frame was based on the hospital’s general ledger numbers assigned to individual pregnant patients and their newborns. The women studied were of similar socioeconomic status, taking into account income, education, occupation and place of residence, and remained at comparable levels throughout the study. All children were born from uncomplicated and full-term pregnancies (between 37 and 42 weeks of gestation). The exclusion criteria for the study were as follows: delivery before 37 weeks of gestation, pregnancy-related medical comorbidities, a severe birth status of the newborn and a lack of written maternal consent. A standardized questionnaire was administered to mothers with items regarding their family history of allergy; perinatal risk factors for impaired intestinal colonization of the newborn; the method of feeding the newborn, including anthropometric data; mode of delivery; course of current pregnancy; and family history of allergy (Table 1).

Patients in the study group were not suffering from any disease during pregnancy, were not taking any medications, and were not suffering from any chronic disease, which was determined on the basis of the interview with the mother. The exception was a group of ten mothers included in the study who received antibiotic therapy only in the third trimester of pregnancy (27–38 weeks of gestation). The medical indications for antibiotic therapy were urinary tract infection (*n* = 7), bronchitis (*n* = 2) and buttock skin abscess (*n* = 1). The patients were treated with oral amoxicillin/clavulanate (875/125 mg) for 7 to 10 days.

**Figure 1:**
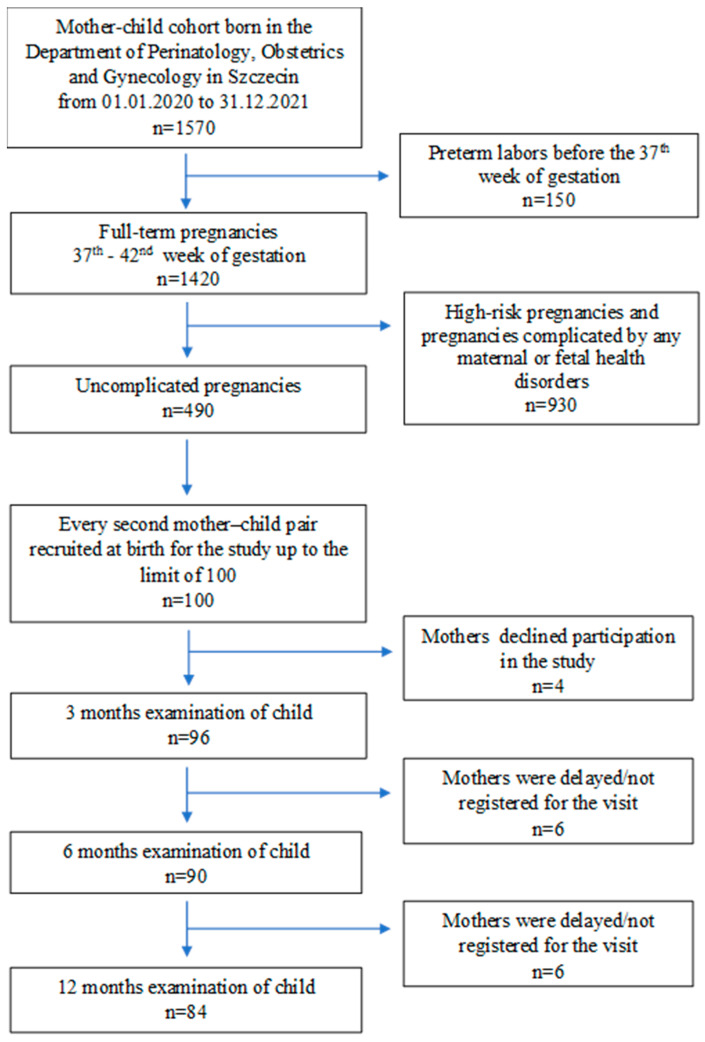
Flow chart of recruitment of mother–newborn pairs and data collection process.

A flow chart of the recruitment of mother–child pairs and the data collection process is shown in Figure 1.

In the infant group, some infants (*n* = 51) received a probiotic preparation. The administered probiotics were preparations containing *L. rhamnosus* GG bacteria, which, next to bacteria of the genus *Bifidobacterium* and *Saccharomyces boulardii* yeast, are the most commonly used agents in humans. Thanks to their antibacterial and anti-inflammatory properties, they contribute to the development of food tolerance, which makes them the first-choice products for infants. Infants involved in the study received a probiotic preparation of lyophilized live cultures of *L. rhamnosus* GG at a concentration of 5 × 10^9^ in five drops of the preparation; of these, 28 infants used the probiotic from 2 weeks to 3 months of age, 16 infants from 3 to 6 months of age and 7 infants for 1 month at 12 months of age. The probiotic was used daily at a dose of five drops. The main indications for use were bloating, gas and mucus in the stool.

### 2.2. Medical Assessment of Children

All infants were examined by a pediatrician, and anthropometric data were collected at birth. The examination of the infants was repeated during their pediatrician’s visit at 0 (i.e., at birth), 3, 6 and 12 months of the child’s life, taking into account the influence of infectious diseases in the early childhood period requiring antibiotic therapy and the method of feeding (i.e., breastfeeding or formula feeding).

A Detecto PD200 medical scale (DETECTO Cardinal Scale Manufacturing Co. 203 E. Daugherty, Webb City, MO 64870, USA) was used to digitally measure weight, height and BMI.

### 2.3. Analysis of Indirect Markers of Intestinal Permeability and Chronic Inflammatory Bowel Disease

At each visit, a sample of the child’s stool was collected for the determination of dysbiosis and intestinal permeability parameters. For this purpose, the zonulin concentration was measured in the childrens’ stool samples by means of ELISA (immunoenzymatic assay) using the “sandwich kit” (sandwich ELISA double-binding assay); the concentrations of LPS and occludin were also determined using ELISA and a sandwich ELISA double-binding assay, respectively. As a cautionary measure, when considering the measurement of serum zonulin as a marker of mucosal barrier integrity, other markers were tested [13]. The determinations of the studied parameters were performed via the immunoenzymatic method using ready-made reagent kits: Lipopolysaccharide (LPS)—LPS ELISA Kit (EIAab), Human Occludin (OCLN) ELISA Kit (EIAab) and Human Zonulin ELISA Kit (Cat. No E3704Hu) from Bioassay Technology Laboratories (BT LAB) (sensitivity: 0.13 ng/mL standard curve range: 0.3–90 ng/mL).

### 2.4. Sample Preparation for the Determination of Lipopolysaccharide (LPS), Zonulin and Occludin Levels

Fecal samples were thawed at refrigerator temperature and then at room temperature. Around 0.3–1.0 g of feces was weighed into a sterile Falcon tube (15) mL. To each tube, 5 mL of phosphate buffered saline (PBS) buffer (pH 7.4), cooled to 4 °C, was added. The samples were mixed thoroughly until homogeneous and then subjected to two cycles of freezing (12–18 h at −20 °C) and thawing (6 h at 4 °C). After the second cycle, the thawed samples were centrifuged at 5000× *g* for 10 min. The resulting supernatant was successively filtered through membrane filters with pore sizes of 0.45 m and 0.22 m. After filtration, the samples were separated into 2 Eppendorf tubes and stored at −80 °C until testing.

### 2.5. Performance Characteristics for Assay Procedure

The ELISA parameters used to determine the zonulin concentration were as follows:

- Sensitivity 0.13 ng/mL; standard curve range: 0.3–90 ng/mL;
- Precision: intra-assay precision—three samples of known concentration were tested on one plate to assess intra-assay precision.
- Inter-assay precision—three samples of known concentration were tested in separate assays to assess inter-assay precision. CV(%) = SD/mean × 100
- Intra-assay: CV < 8% Inter-Assay: CV < 10%
- Specificity: this sandwich kit was for the accurate quantitative detection of human zonulin; haptoglobin (also known as HP).

The ELISA parameters used to determine the concentration of LPS were as follows:

- Sensitivity: the minimum detectable dose of LPS was typically less than 0.03 EU/mL. The sensitivity of this assay, or lower limit of detection (LLD) was defined as the lowest protein concentration that could be differentiated from zero. We determined the mean O.D. value of 20 replicates of the zero standard added, according to their three standard deviations.
- Detection range: 0.078–5 EU/mL. The standard curve concentrations used for the ELISAs were 5 EU/mL, 2.5 EU/mL, 1.25 EU/mL, 0.625 EU/mL, 0.312 EU/mL, 0.156 EU/mL and 0.078 EU/mL. Specificity: this assay had high sensitivity and excellent specificity for the detection of LPS. No significant cross-reactivity or interference was observed.
- Precision: Intra-assay precision (precision within an assay): three samples of known concentration were tested twenty times on one plate to assess intra-assay precision. Intra-assay CV: ≤5.3%.

For the occludin assay, the ELISA parameters were: intra-assay precision: CV% < 8%. Three samples of known concentration were tested twenty times on one plate to assess inter-assay precision. Inter-assay precision (precision between assays): CV% < 10%. Three samples of known concentration were tested in twenty assays to assess the following. Sensitivity: 0.039 ng/mL; detection Range: 0.156 ng/mL–10 ng/mL. Specificity: this kit recognized human OCLN in samples. No significant cross-reactivity or interference between Human OCLN and analogues was observed.

### 2.6. Statistical Analysis

Statistical analysis was performed using STATA 11 statistical software (StataCorp LLC 4905 Lakeway Drive, College Station, TX 77845-4512, USA; license number 30110532736). In all tests performed, statistically significant differences were considered as those with a *p*-value < 0.05. A significance level of *p* = 0.051–0.099 was designated as a trend at the limit of statistical significance. The normality of data distributions was tested using the Kolmogorov–Smirnov test. If *p* < 0.05 (i.e., there was no normal distribution of data), a Mann–Whitney or Kruskal–Wallis test was used. Correlations were calculated using Spearman’s rank test. Based on the analysis, the distribution was found to be non-normal; therefore, ANOVA was not used.

## 3. Results

In the group of pregnant women, the mean age was 31.02 years (median: 31); the mean weight and BMI before delivery were 68.27 kg (median: 65) and 24.55 (median: 22.74), respectively; and the mean weight and BMI after delivery were 82.05 kg (median: 80) and 29.50 (median: 28.53), respectively. All pregnancies were term, and the mean gestational age was 38.4 weeks. Of the women included in the study, 44 (44%) gave birth naturally and 56 (56%) had a cesarean section. The median birth weight was 3300 g (Table 1).

In this study, we confirmed the presence of zonulin, occludin and LPS in the feces of newborns and neonates and determined their concentrations at 0, 3, 6 and 12 months of age. The median LPS concentrations at 0, 3, 6 and 12 months of age were 43.16, 37.61, 36.99 and 48.29 pg/g, respectively. The median occludin concentrations at 0, 3, 6 and 12 months of age were 2.36, 2.25, 2.17 and 2.17 ng/g, respectively, whereas the medians zonulin concentrations at 0, 3, 6 and 12 months of age were 74.14, 68.84, 63.41 and 43.88 ng/g, respectively (Table 2).

In the correlational research between continuous variables, the strongest interrelationships of the studied fractions were observed during the first 3 months of the infants’ lives. During this period, higher values of occludin and zonulin were most often accompanied by higher values of LPS, and this relationship was significant. Similarly, higher zonulin values were accompanied by higher occludin values. Significant differences were found in the analysis of the concentration values of the individually determined components of infant feces. LPS levels measured after birth showed a statistically significant difference compared to the LPS levels recorded at 3 and 6 months of age. Similarly, the concentrations of occludin at birth showed a statistically significant difference when compared to the occludin concentrations reported at 3 and 6 months of age. After delivery, zonulin concentrations, similarly to occludin, showed a statistically significant difference when compared with zonulin determined at 3 and 6 months of age.

**Figure 2:**
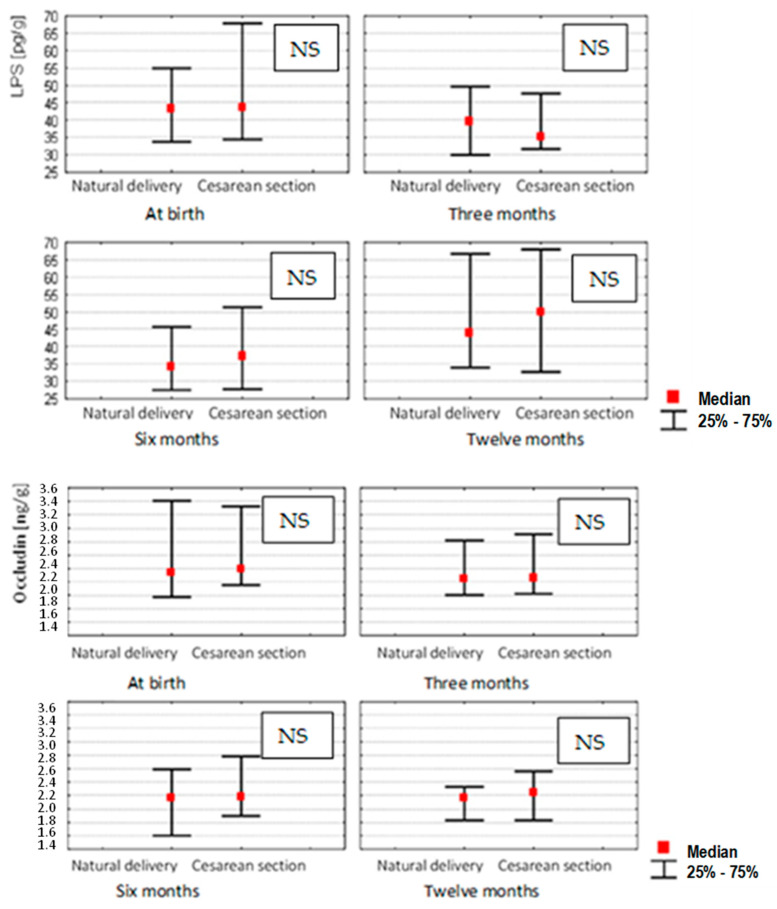

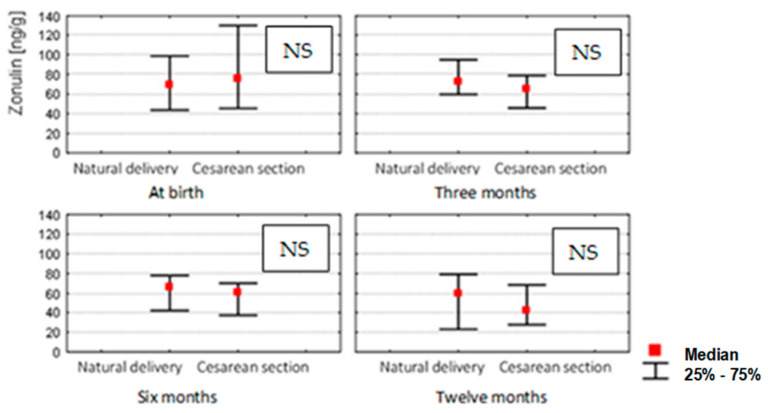
Stool concentrations of studied parameters for 0 (*n* = 100), 3 (*n* = 96), 6 (*n* = 90) and 12 (*n* = 84) month-old children depending on the method of delivery (Mann–Whitney U test and Kruskal–Wallis test; *p*-value ≤ 0.05 was considered statistically significant. NS: non-significant).

Next, we analyzed how the ranges based on quartiles from a total of three tests performed at 0, 3, 6 and 12 months for LPS, occludin and zonulin correlated with the mode of delivery. No significant differences were found in the group of women who had natural deliveries compared to those whose gave birth by caesarean section (Figure 2).

In the analysis of dependent variables presented above, their correlation was not influenced by factors such as the women’s level of education, smoking or the presence of allergies in their family history. The method of feeding did not significantly affect the changes in the concentrations of the determined fractions. In the group of mothers who breastfed their children from birth, the concentrations of LPS were not significantly low in comparison to non-breastfeeding mothers. Occludin and zonulin showed a similar relationship (Table 3).

**Figure 3:**
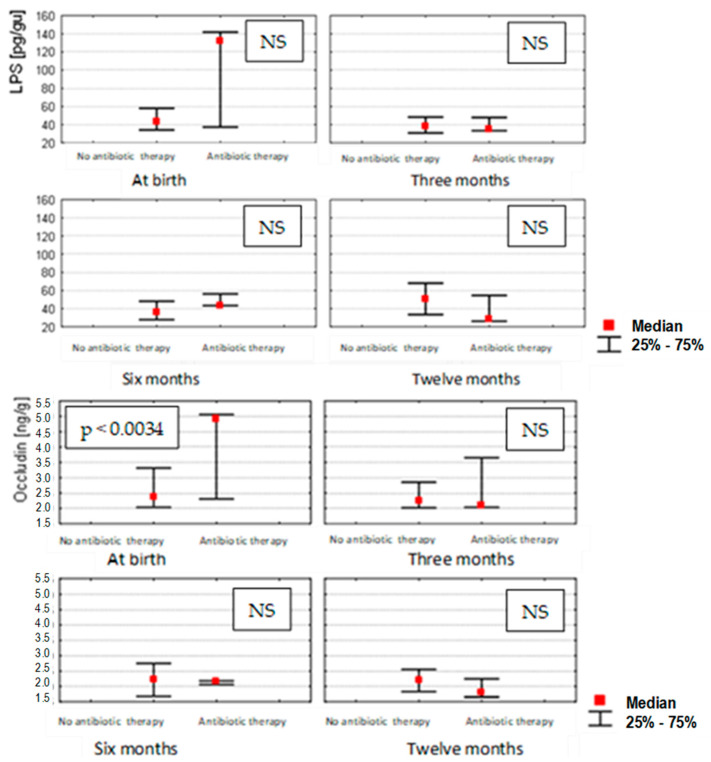

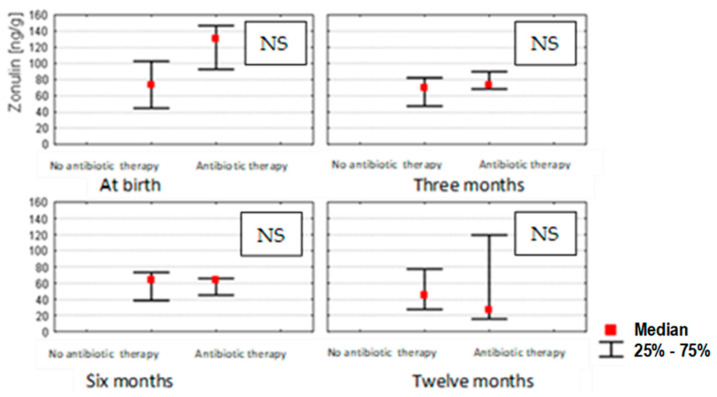
Stool concentrations of studied parameters at birth (*n* = 100), in 3 (*n* = 96), 6 (*n* = 90) and 12 (*n* = 84) month-old children depending on exposure to antibiotic therapy during pregnancy (Mann–Whitney U test and Kruskal–Wallis test; a *p*-value *≤* 0.05 was considered statistically significant. NS: non-significant).

The effect of antibiotics administered to pregnant women during their third trimester of pregnancy on the zonulin, occludin and LPS concentrations in the stools of infants changed during the different months that were studied. Statistical correlations were found (*p* < 0.0034) only in the case of occludin measured at birth (0 month) in children from mothers undergoing antibiotic therapy, in whom occludin concentrations were significantly higher. In the subsequent months of the infants’ lives, such correlations were no longer observed for occludin, zonulin or LPS (Figure 3).

**Figure 4:**
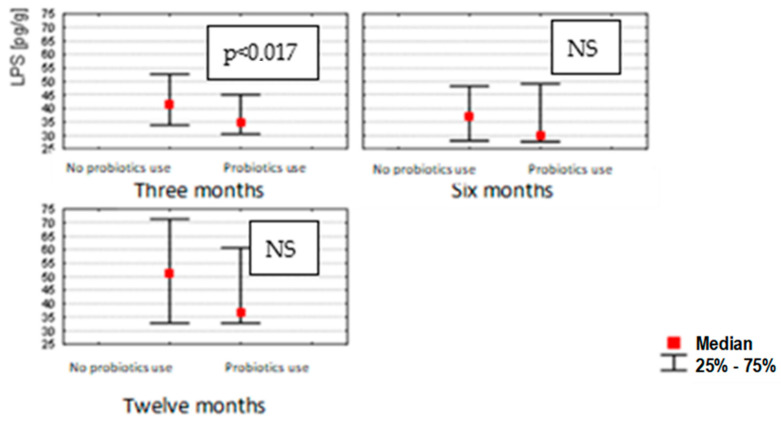
LPS concentrations in stools of 3 (*n* = 96), 6 (*n* = 90) and 12 (*n* = 84) month-old children, depending on probiotic application after birth. (Mann–Whitney U test and Kruskal–Wallis test; a *p*-value *≤* 0.05 was considered statistically significant. NS: non-significant).

The use of probiotics in infants resulted in a decrease in the concentration of LPS only at 3 months (*p* < 0.017); probiotics did not affect the concentrations of zonulin or occludin at 0, 6 and 12 months (Figure 4).

An analysis of the effect of the pregnant women’s weight before and after delivery on zonulin, occludin and LPS concentrations at 0, 3, 6 and 12 months showed no correlation in overweight women (BMI 25–29.9) before delivery, and no correlation was observed in those with obesity (BMI > 30) or of a normal weight after delivery.

## 4. Discussion

### 4.1. LPS, Occludin and Zonulin Stool Concentrations in Infants during the First Year of Life

In this study, we measured LPS, occludin and zonulin concentrations in the feces of newborns after birth (0 months) and during the first 3, 6 and 12 months of life. Repeated biomarker studies in subsequent months are important in determining trends, and more reliably reflect the status of intestinal barrier integrity. According to Vojdani et al. [14], due to concentration fluctuations, a single measurement of zonulin levels is not recommended for the assessment of intestinal barrier integrity. In the literature, biomarkers measured in blood serum and stools, such as zonulin and calprotectin [15], are most commonly used to assess intestinal permeability, whereas there are no studies defining the evolution of occludin and LPS concentrations in the meconium and feces of children during the first year of life. In our study, the strongest correlations between the investigated fractions were observed during the first 3 months of the infants’ lives. Higher values of occludin and zonulin were associated with higher levels of LPS; in addition, higher values of zonulin were accompanied by higher levels of occludin. In their study, Khasanova et al. [16] examined zonulin concentrations in the feces of neonates born prematurely and at term. They found that the zonulin concentrations in the stools of newborns with lower body weight were higher compared to children born with higher body weight, which may reflect increased intestinal permeability resulting from intestinal immaturity at an earlier stage of development. It is likely that increased intestinal permeability may occur during the first period of neonatal life, which is correlated with an increased degree of endoxemia expressed by higher LPS concentrations, as indirectly indicated by the results obtained in our study.

Our results indicate that during the first period of neonatal life, there is increased intestinal permeability, causing the temporary disorganization of proteins that form the tight junctions of the colonic epithelium, which is correlated with an increased degree of endoxemia, expressed as higher LPS values.

According to the results of Kaczmarczyk et al. [17], intestinal permeability in the first month of life, measured via zonulin levels, is lower than in the later period, suggesting that the possibility for antigen translocation during the early period after birth is limited. With changes in dietary habits and the maturation of the intestinal barrier over the next 6 months, biomarker concentrations are reduced, as confirmed in our study. The relationship of dietary changes with intestinal permeability was observed in an animal model. Martínez-Oca et al. [18] found significantly higher levels of ZO-1 and occludin in the colonic epithelium of rats with concomitant thinning due to the introduction of a restrictive diet. On the other hand, Zak-Golab et al. [19] showed a correlation between total bacterial content and serum zonulin levels, suggesting that the gut microbiota may cause increased zonulin levels, which subsequently leads to abnormal intestinal permeability to endotoxin (LPS) and the consequent microinflammation observed in obesity. In a study by Kaczmarczyk et al. [17], an initial trend of increasing zonulin concentrations in the stools of infants was observed up to 6 months after birth and was followed by stabilization at high levels between about 6 and 12 months. A close correlation exists between the gut microbiota during early life, intestinal function and the metabolic health of children [20].

### 4.2. LPS, Occludin and Zonulin Stool Concentrations in Infants Depending on the Mode of Delivery

Although the development of the microbiota of neonates born vaginally is quite different from that of children born by caesarean section [21], our analysis of the mode of termination of pregnancy in relation to the selected stool biomarkers in the subsequent months of the children’s lives did not show any significant differences. In contrast, a study by Łoniewska et al. [22] found higher concentrations of zonulin in the stools of newborns on day 7 after birth in a group of children born by caesarean section compared to those delivered naturally. Elevated fecal concentrations of other intestinal permeability biomarkers, such as calprotectin, have been observed in association with cesarean sections [23]; however, to our knowledge, there have been no studies investigating LPS and occludin concentrations in relation to the mode of delivery. Although bacterial diversity and the extent of gut microbiota colonization in the first three months of life are significantly associated with the mode of delivery, the significant differences disappear after 6 months. In another study of 6 month-old infants, the patterns of colonization were almost the same for both modes of termination [24], which agrees well with our results. On the other hand, a critical assessment in light of the existing evidence for a causal relationship between cesarean delivery and neonatal dysbiosis was presented by Stinson et al. [25].

### 4.3. LPS, Occludin and Zonulin Stool Concentrations in Infants Depending on Other Factors

In another study, several factors, including antibiotic therapy, an increase in BMI of more than 5.7 during pregnancy and natural childbirth, were found to contribute to increased intestinal permeability in children during the first two years of life, as indicated by fecal zonulin and calprotectin concentrations [22].

Our study showed that factors such as maternal education, smoking and the presence of allergies in the family history did not show any significant influence. Other studies have shown that cigarette smokers have high levels of fecal zonulin, which is associated with significantly increased intestinal permeability [26]. However, these studies did not analyze other markers of impaired intestinal permeability and fecal battery endoxemia, such as occludin and LPS concentrations [26].

### 4.4. LPS, Occludin and Zonulin Stool Concentrations in Infants Depending on the Mode of Feeding

In our study, the median concentrations of LPS, zonulin and occludin in stools were not significantly different between children born via caesarean section and those born via vaginal birth.

It is possible that exclusive breastfeeding may be associated with the rapid functional maturation of the neonatal intestinal barrier. It has been shown that breastfeeding can partially restore abnormalities in the infant’s intestinal microbiota caused by cesarean section; in particular, it can promote a reduction in the abundance of bifidobacteria [20]. The effect of feeding methods (breastfeeding vs. formula feeding) was investigated in another study, and the results showed higher calprotectin concentrations in the stools of breastfed infants; however, fluctuations in the concentrations of other biomarkers were not studied [27]. In addition, the early inclusion of complementary nutrition may be associated with future childhood obesity [10]. In a study by Li et al. [27] of a group of 105 healthy children, significantly higher calprotectin concentrations were found in breastfed children compared to non-breastfed children. At the same time, a negative correlation of calprotectin in relation to age in the first 0–5 months of life was observed, regardless of the method of feeding. The above studies confirmed the role of feeding in the modulation of intestinal marker concentrations in healthy children. However, other biomarkers of intestinal permeability in children’s stools, including occludin and zonulin, have not been studied.

### 4.5. LPS, Occludin and Zonulin Stool Concentrations in Infants Depending on Antibiotic Use

In the group of pregnant women who received antibiotic therapy, higher median values for occludin concentrations at birth were observed, with no change in the median values for occludin, zonulin, and LPS concentrations in subsequent months of infant life. In a study by Nevado et al. [28], performed on mice, it was found that neomycin and bacitracin-induced microbiota depletion reduced intestinal permeability and increased the expression of not only occludin but also other markers, including ZO-1 and JAM-A in the ileum and ZO-1, claudin-3 and claudin-4 in the colon. There have been reports showing a positive correlation between fecal zonulin concentrations in children and antibiotic therapy during pregnancy [29]. Prenatal exposure to antibiotics is not only for therapeutic purposes; antibiotics may also be used intrapartum as prophylaxis in GBS-positive women and intraoperatively during cesarean sections [1,21].

### 4.6. LPS, Occludin and Zonulin Stool Concentrations in Infants Depending on Probiotic Use

In our study, probiotic-treated infants were found to have lower median LPS levels at 3 months of age, with no apparent differences in median zonulin and occludin concentrations at 0 and 6 months of age. Although there have been reports on the beneficial effects of probiotics on serum zonulin levels [30], there are no data on the effects of probiotics on other biomarkers. There is also a lack of data in the literature regarding the nature of the reduction in LPS concentrations in the stools of probiotic-fed children. In a study conducted in rats, probiotics effectively inhibited LPS-induced autophagy, indicating that probiotic involvement may be associated with enteroprotection against LPS-induced intestinal epithelial toxicity [31].

In a review paper published by Skonieczna-Żydecka et al. [29] concerning the effect of probiotics on symptoms, intestinal microbiota and inflammatory markers in infantile colic, a causal relationship between probiotic use and children’s microbiota and immune systems was not clearly confirmed.

### 4.7. LPS, Occludin and Zonulin Stool Concentrations in Infants Depending on Maternal Gestational Weight Gain

No changes were observed in the median concentrations of zonulin, occludin or LPS at 0, 3, 6 and 12 months of infancy relative to the change in body weight of pregnant women before and after delivery. In a study by Łoniewska et al. [22], a negative correlation was noted between an increase in BMI during pregnancy and maternal serum zonulin concentrations; no effects were reported on its concentrations in the stools of newborns at 7 days of life. However, it seems that it is not the BMI value during pregnancy, but rather the diet leading to obesity, that has a significant effect on the microbiota of children. The maternal diet in the periconceptional period and during pregnancy is therefore a critical and modifiable element that significantly affects the health of the child [32].

One limitation of the present study is the use of a relatively small group from one center. Enlarging the study group would allow for more definitive conclusions, as well as multivariate analyses. In addition, preterm births were not included in the study design; however, in such cases, the effects of prematurity on inflammatory parameters, biomarkers of intestinal permeability and intestinal dysfunction in newborns could be more accurately determined. The high sensitivity and relative simplicity of the direct sandwich ELISA makes it a frequently used assay. The main problem with this method is that there is no single universal procedure that works for the testing of different types of materials. All parameters that are essential for the proper implementation of the test are highly specific and strongly dependent on the reagents used. Therefore, before using the ELISA technique, the optimization of these parameters is required. Depending on the ELISA format used, the optimization process can be quite complicated. The low availability of specific antibodies on the market should also be considered as a definite disadvantage of this test.

## 5. Conclusions

Perinatal factors related to intestinal permeability are important during the first 3 months of infant life. However, in our study, the mode of delivery had no influence on the parameters of infant intestinal leakage during the first year of life.

No changes were observed in the median concentrations of LPS, zonulin or occludin in the stools of either breastfed or exclusively formula-fed children.

A short-term increase in occludin concentrations after delivery in the stools of children from mothers undergoing antibiotic therapy indicated a negative but reversible influence of intrapartum antibiotics on the intestinal integrity of children in the perinatal period.

Probiotic therapy seems to have a positive effect by reducing intestinal inflammation in children during the first 3 months of life; however, a study on a larger population is necessary to confirm this theory. LPS concentrations at 3 months have nothing to do with the tightness of the intestines of the later measured periods of the infants’ lives.

## Figures and Tables

**Table 1:** Clinical characteristics of the analyzed group (*n* = 100).

Variables	Median (Min–Max)
Age (years)	31.02 (27–34) *
Maternal weight at visit 1 (kg)	65.00 (57–72) *
Maternal BMI at visit 1 (kg/m^2^)	22.74 (20.9–26.5) *
Maternal weight during labor (kg)	80.00 (70.5–88.5) *
Maternal BMI during labor (kg/m^2^)	28.53 (26–31.2) *
Parity	2 (1–2) *
Gestation age (weeks)	39.00 (38–39) *
Vaginal delivery *n* (%)	44 (44)
Cesarean section *n* (%)	56 (56)
Baby birth weight	3300 (2990–3565) *
APGAR score at 5th min.	10 (all 10) *

BMI—body mass index; APGAR—ten point scale; * Data are median (interquartile range) values.

**Table 2:** Values of fecal lipopolysaccharide (LPS), occludin and zonulin in the examined children (nonparametric Mann–Whitney test).

	*n*	Q25	Median	Q75
LPS (pg/g)				
0 months (at birth)	100	34.12	43.16	59.30
3 months	96	31.20	37.61	48.01
6 months	90	27.75	36.99	48.36
12 months	84	32.90	48.29	67.86
occludin (ng/g)				
0 months (at birth)	100	2.05	2.36	3.39
3 months	96	2.02	2.25	2.88
6 months	90	1.68	2.17	2.63
12 months	84	1.83	2.17	2.53
zonulin (ng/g)				
0 months (at birth)	100	45.24	74.14	106.05
3 months	96	48.75	68.84	82.40
6 months	90	38.50	63.41	72.27
12 months	84	25.93	43.88	77.12

LPS—Lipopolysaccharide.

**Table 3:** Analysis of dependent variables.

Variable	Month	LPS	Occludin	Zonulin
		*p* *	*p* *	*p* *
Mother’s education	0	0.78	0.54	0.90
	3	0.90	0.25	0.93
	6	0.92	0.87	0.29
	12	0.59	0.74	0.46
Cigarette smoking	0	0.61	0.71	0.56
	3	0.86	0.68	0.73
	6	0.53	0.68	0.48
	12	0.79	0.56	0.77
History of allergy	0	0.66	0.28	0.55
	3	0.50	0.10	0.41
	6	0.61	0.60	0.73
	12	0.73	0.67	0.43
Breastfeeding	0	0.77	0.35	0.51
	3	0.88	0.56	0.45
	6	0.54	0.28	0.26
	12	0.65	0.18	0.83
Formula feeding	0	0.49	0.55	0.26
	3	0.45	0.98	0.23
	6	0.47	0.50	0.74
	12	0.63	0.94	0.44

*p* *—Spearman’s rank correlation coefficient.

## Data Availability

Not applicable.
